# Optical Absorption Exhibits Pseudo-Direct Band Gap of Wurtzite Gallium Phosphide

**DOI:** 10.1038/s41598-020-64809-4

**Published:** 2020-05-13

**Authors:** Bruno C. da Silva, Odilon D. D. Couto, Hélio T. Obata, Mauricio M. de Lima, Fábio D. Bonani, Caio E. de Oliveira, Guilherme M. Sipahi, Fernando Iikawa, Mônica A. Cotta

**Affiliations:** 1 Institute of Physics “Gleb Wataghin”, University of Campinas, 13083-859 Campinas, São Paulo, Brazil; 2 Materials Science Institute, University of Valencia, 22085, 46071, Valencia, Spain; 3 São Carlos Institute of Physics, University of São Paulo, 369, 13566-590 São Carlos, SP Brazil

**Keywords:** Semiconductors, Nanowires, Nanoscale materials, Electronic properties and materials, Nanowires, Raman spectroscopy

## Abstract

Definitive evidence for the direct band gap predicted for Wurtzite Gallium Phosphide (WZ GaP) nanowires has remained elusive due to the lack of strong band-to-band luminescence in these materials. In order to circumvent this problem, we successfully obtained large volume WZ GaP structures grown by nanoparticle-crawling assisted Vapor-Liquid-Solid method. With these structures, we were able to observe bound exciton recombination at 2.14 eV with FHWM of approximately 1 meV. In addition, we have measured the optical absorption edges using photoluminescence excitation spectroscopy. Our results show a 10 K band gap at 2.19 eV and indicate a weak oscillator strength for the lowest energy band-to-band absorption edge, which is a characteristic feature of a pseudo-direct band gap semiconductor. Furthermore, the valence band splitting energies are estimated as 110 meV and 30 meV for the three highest bands. Electronic band structure calculations using the HSE06 hybrid density functional agree qualitatively with the valence band splitting energies.

## Introduction

In order to generate white-light based on red-green-blue (RGB) model from high efficiency light emitting diodes, a green source with considerable efficiency is still required. Different materials have emerged as candidates to overcome this barrier; among them, in III-V compounds, hexagonal Gallium Phosphide (GaP) has attracted attention as a potential phosphor-free solid state green/yellow emitter^1–3^. The Wurtzite (WZ) phase of Gallium Phosphide has been studied in the last few years due to the novel properties obtained by controlling the crystal structure, and it has been predicted as a direct (or pseudo-direct) band gap semiconductor, with emission in the green/yellow spectral range^4–6^.

However, the exact value of the band gap as well as other parameters, such as excitonic emissions and valence band splitting, are still under debate in literature, which reports distinct optical results^1,2,7–11^. For instance, the predicted value for the band gap of WZ GaP is different between several reported works and shows disagreement with the observed values^4–6,12^. The difficulty of studying band-to-band emissions of the hexagonal phase originates from the small oscillator strength of the lowest energy transition, leading to weak photoluminescence (PL)^2,8–12^. Although WZ GaP presents its lowest energy electronic transition at the Γ point, the material behaves as a pseudo-direct band gap semiconductor, due to the weak oscillator strength at this point (forbidden fundamental electronic transition). This behavior has been pointed out as a possible reason for the absence of the fundamental band-to-band absorption edge in previous photoluminescence excitation (PLE) studies^13,14^. Nevertheless, despite the expected pseudo-direct band gap, Assali *et al*. were able to observe excitonic recombinations, such as bound excitons^2^. All these features show that the full understanding of the optical properties of this material is yet to be achieved. Therefore, in order to design new optoelectronic devices based on hexagonal phase GaP, further knowledge about the optical properties of this material is required.

Here, we have probed the electronic band structure of the WZ phase in GaP using Photoluminescence Excitation (PLE) Spectroscopy. In order to enhance the PL signal detected in PLE, large volume WZ GaP structures were successfully grown, allowing us to measure the lowest energy band-to-band absorptions edges of this material. Our results indicate a threshold of 2.19 eV at 10 K for the fundamental band gap. Moreover, higher energy absorptions related to the valence band splitting were also observed.

## Experimental Methods

The GaP structures were grown by Chemical Beam Epitaxy using Au nanoparticles as catalyst. The growth conditions used here were the same reported previously^10^ in order to promote the growth of large and asymmetric GaP nanowires in the WZ phase. However, longer growth times (up to 6 h) were employed in this case. Secondary electron imaging was carried out in a FEI Inspect F50.

The optical analysis were performed using photoluminescence spectroscopy (PL), micro-PL (µ-PL) and photoluminescence excitation (PLE) spectroscopy at low temperature and micro-Raman (µ-Raman) at room temperature. For µ-PL and µ-Raman the data were collected in the backscattering geometry using 50x objective lens (laser spot diameter of ~ 2 µm) and a solid state laser emitting at 488 nm as excitation source. In these experiments, the GaP structures were transferred to a Si/SiO_2_ substrate. The μ-PL and μ-Raman signals were detected by a Si CCD detector in a single monochromator with 600 and 1800 gr/mm grating, respectively. For macro-PLE analysis, a Xenon lamp coupled to a single monochromator was used as excitation light. The luminescence detection was performed by a 0.75 m double monochromator with 1200 gr/mm grating and a GaAs-cathode photomultiplier.

The band structure was calculated using Density Functional Theory (DFT), as implemented in the Vienna ab initio simulation package (VASP)^15,16^, version 5.4.4. In order to obtain realistic gap energies, we used the Heyd-Scuseria-Ernzerhof hybrid functional (HSE06)^17^ including spin-orbit corrections, as done for III-V zinc-blend alloys^18,19^.

## Results and Discussion

**Figure 1:**
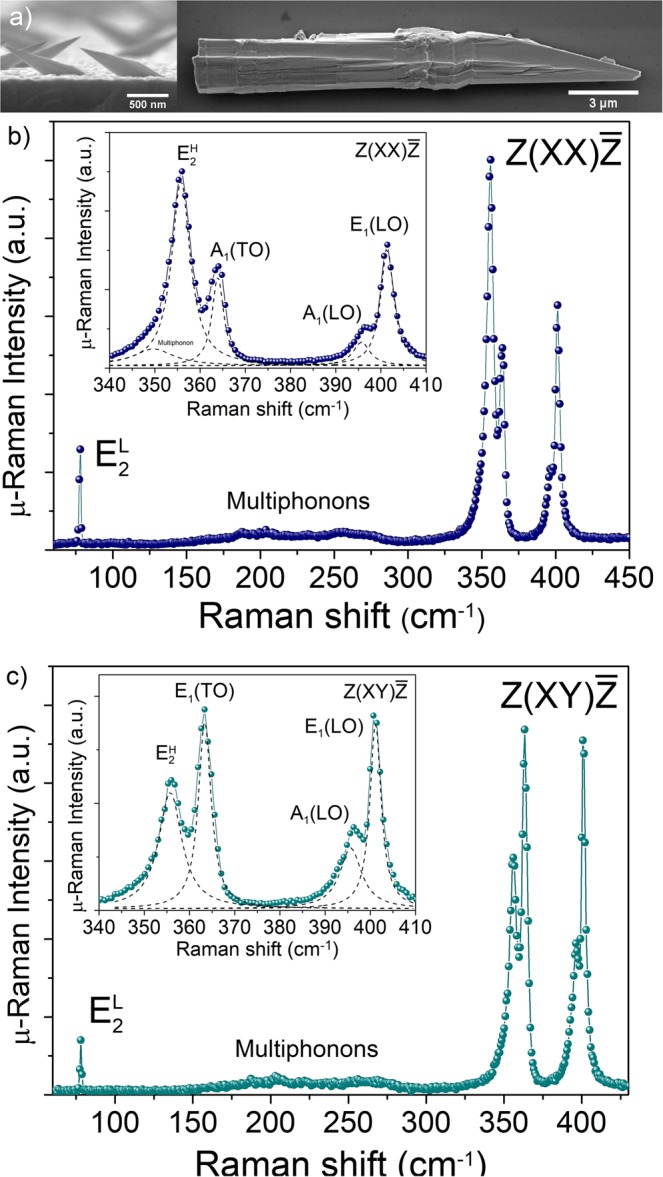
(**a**) Side-view SEM image of as-grown asymmetric WZ GaP nanowire and top-view SEM image of GaP structure grown for 6 hours and transferred to Si substrate. (**b**) and (**c**) Room temperature Raman spectra performed in the $Z(XX)\overline{Z\,}\,$and $Z(XY)\overline{Z\,}\,$configurations, respectively, showing the ${E}_{2}^{L}$ mode characteristic of the hexagonal phase. Insets show the spectra around the optical phonons. The deconvoluted modes are shown in dashed lines.

Figure 1a shows scanning electron microscopy (SEM) images of as-grown GaP asymmetric NWs (grown for 1 h) and the GaP structure (grown for 6 h) transferred to a Si substrate. Large volume and asymmetric morphologies, similar to those previously reported^10^, are observed. On average, the as-grown structures have a length of approximately 40 µm. In order to probe the crystal structure, we used room temperature μ-Raman spectroscopy using two line polarization configurations: $Z(XY)\overline{Z\,}\,$and $\,Z(XX)\bar{Z}$. Here $X$ and $Y$ are the light polarization direction of the excitation and detection. In our experiment $X$ lays along the basal plane and $Y$ along the c-axis of the WZ crystal. The directions $Z\,$and $\bar{Z}$ are the incident and scattered light directions, respectively, along the perpendicular direction of X and Y axis.

The Raman spectra depicted in Fig. 1b show the characteristic Raman active vibrational mode ${E}_{2}^{L}$ of the WZ GaP at 78.1 cm^−1^^20^. Other Raman allowed modes in $Z(XX)\bar{Z}$ configuration are ${E}_{2}^{H}$ at 355.8 cm^−1^ and A_1_(TO) at 363.4 cm^−1^, (see Fig. 1b), while the $Z(XY)\bar{Z}\,$configuration provides only E_1_(TO) mode at 363.4 cm^−1^, (see Fig. 1c). The ${E}_{2}^{H}$ peak in $Z(XY)\overline{Z\,}\,$is observed due to the relaxation of selection rules; the forbidden modes assigned as E_1_(LO) at 401.4 cm^−1^ and A_1_(LO) at 395.7 cm^−1^ are also observed. Multiphonon scattering is also observed between 150–300 cm^−1^ in the Raman spectra. Therefore, the μ-Raman scattering results show clear evidence of the WZ phase in the GaP structure, since zinc-blend (ZB) GaP presents only two optical modes. Moreover, the peak at 78.1 cm^−1^, i.e., ${E}_{2}^{L}$ mode is absent in ZB phase. The pronounced forbidden LO modes presented in Fig. 1b,c are most likely caused by resonance effects. The 488 nm (2.54 eV) laser line is very close to the high energy band transition as demonstrated below in our PLE spectra. Recently, Panda *et al*. have shown the use of this resonance effect to investigate the electronic transitions in WZ GaP^20^. It is important to note that the lateral dimension (d) of the WZ GaP structure discussed here is much larger than usual nanowires and, since d » λ, (λ is the wavelength of the laser spot) we rule out antenna effects^21^. Similar enhancement has also been observed in other polar semiconductors such as in GaAs^22^, InAs^23^, and CdS^24^. In most cases, this resonance effect leads the forbidden LO modes to reach the same order of magnitude than the allowed modes at resonance^20,22–24^. The LO phonons involve uniform displacements of charged atoms, leading them to be accompanied by a macroscopic electric field. Consequently, it is expected that electrons couple to these LO phonons in polar semiconductors, a phenomenon described by the Fröhlich interactions^25^. This breakdown of the Raman selection rules has been observed for nanostructures as well as for bulk materials^20,22–24,26^. Therefore, we speculate that, in this case, the mechanism behind the LO strengthening could be related to purely bulk effects depending on the excited electronic state, as proposed for CdS^24^.

**Figure 2:**
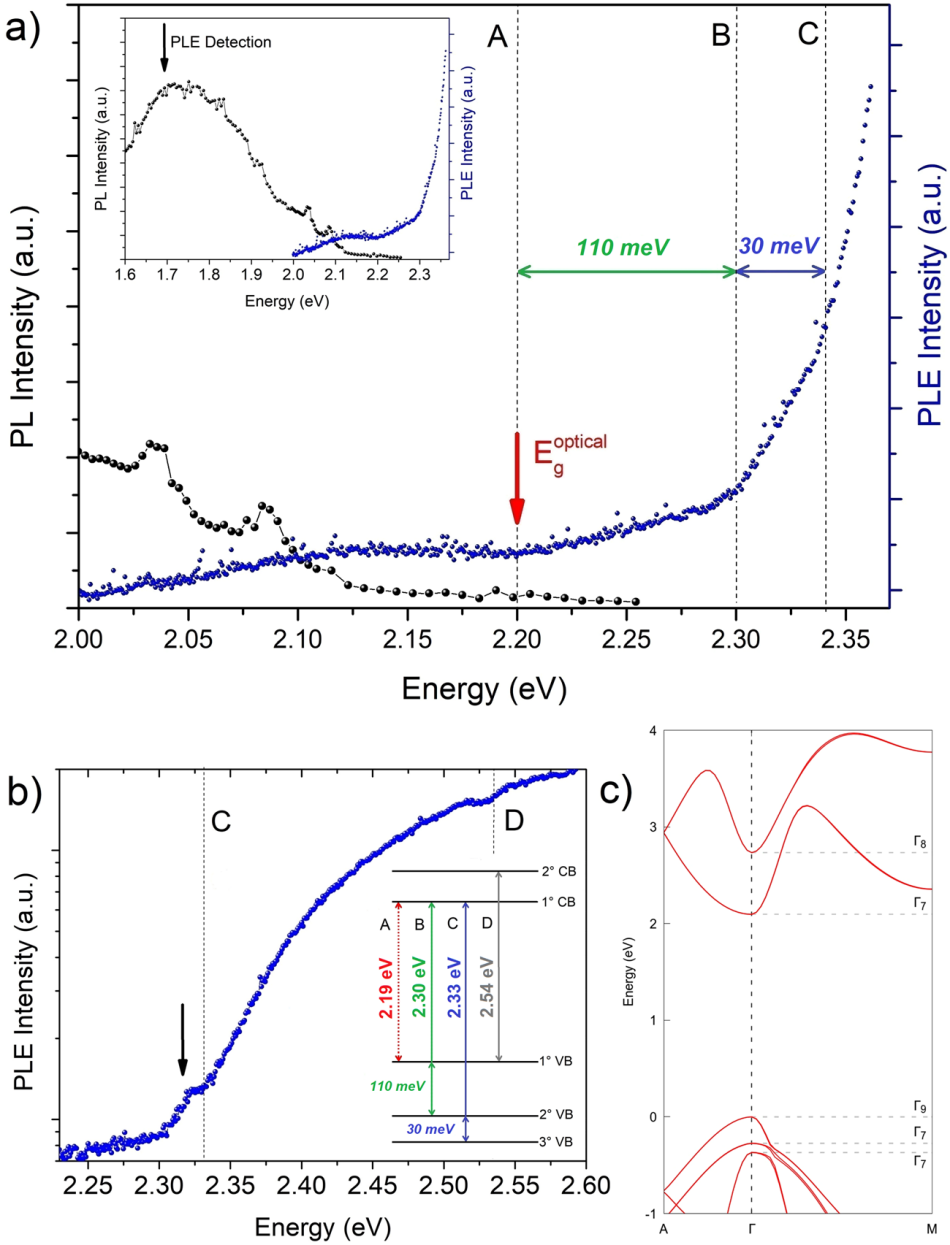
(**a**) PL and PLE spectra of the GaP structures performed at 10 K; dashed lines indicate the absorptions edges labeled *A*, *B* and *C*. Inset: the same PL and PLE spectra showed in (**a**) with broad range. The black vertical arrow indicates the detection position at 1.68 eV, used in PLE measurements. PLE was acquired using the maximum of the Xe lamp single monochromator aperture slit (2 mm) and a long integration time, in order to increase the signal/noise ratio of the ground absorption edges. (**b**) PLE performed with Xe lamp single monochromator aperture slit of 0.75 mm; additional absorption edge was observed at high energy and labeled *D*. The black arrow indicates the excitonic peak observed for transition *C*. Inset: schematic representation of the first absorption edges observed in PLE measurements. Dashed (solid) lines indicate the weak (strong) absorption edges with monotonic (parabolic) increase with energy. (**c**) Electronic band structure of the WZ GaP calculated by DFT method using HSE06 functional, implemented in the Vienna ab initio simulation package (VASP)^15,16^, version 5.4.4.

The electronic band structure has been probed using PL and PLE spectroscopy performed at 10 K, presented in Fig. 2a. The PL spectrum shows strong broad bands at 1.88 eV and 1.68 eV as well as two sharp lines (FWHM ~ 30 meV) at 2.04 eV and 2.09 eV. The same characteristic emission peaks, with similar bandwidth, have been reported for WZ GaP NWs^1,10^. They were observed along the whole large GaP structure and in samples grown in different conditions. The detection of PLE experiments was monitored at 1.68 eV from the broad band and, in order to improve the signal, we have used a large aperture slit in the double-monochromator (2 mm). As we can see in Fig. 2a, a weak absorption starts at 2.00 eV and continues to be observed up to 2.10 eV. This unexpected absorption, observed below the estimated band gap at 2.19 eV^2^, could be associated to impurity absorption. Indeed, impurity based emissions, such as donor-acceptor pair (DAP) recombination at 2.04 eV and 2.09 eV^1,2^, can also be seen in our PL spectrum, shown in the inset of Fig. 2a.

However, the most interesting results in the PLE spectra are the successive absorption edges shown in Fig. 2a: a very weak edge at (2.19 ± 0.02) eV, and two stronger absorptions at (2.30 ± 0.03) eV and (2.33 ± 0.02) eV. The quantitative analysis related to the determination of the optical absorption thresholds is discussed in the Supplementary Information SI.1. These features can be much better observed in Fig. 2b; in this case, the output slit of our Xe lamp single monochromator was reduced from 2 to 0.75 mm in order to increase the spectral resolution. The resulting PLE spectrum shown in Fig. 2b presents an excitonic-like peak at ~ 2.318 eV, that is just below the absorption onset at 2.33 eV, leading to an estimate for the exciton binding energy of 12 meV. Figure 2b also shows an electronic transition at 2.54 eV, which could be related to the transition involving the second conduction band and the top of the valence bands, which is in agreement with the previous result reported by Panda *et al*.^20^ measured using Raman resonance scattering. The electronic transitions observed are summarized in the inset of Fig. 2b.

The fundamental band gap of the WZ GaP nanowires has been experimentally estimated in previous reports in the range 2.09 eV – 2.19 eV, based on PL measurements^1,2,7^. Thus, combining our experimental results with previously reported data, we can conclude that there is a threshold for the absorption, i.e., a band gap at E_g_ = (2.19 ± 0.02) eV at 10 K for the WZ phase, which is in good agreement with the experimental value reported by Assali *et al*.^2^

Moreover, interesting features can be pointed out. First, the lowest band-to-band absorption edge, labeled as *A* in Fig. 2a and in the inset of 2b (dashed lines), is very weak with a monotonic increase in energy. On the other hand, the absorption related to *B* and *C* transitions are much stronger than from *A*, especially for the *C* transition, with the presence of an excitonic-like peak (Fig. 2b). The energy dependence of the absorption spectrum above the excitonic-like peak behaves as a typical three-dimensional (3D) parabolic band transition around the edge^27^. These observations point to a large oscillator strength for the *C* transition.

The optical absorption of semiconductors can be used as a fingerprint for the nature of the bandgap. For instance, the direct allowed transition (typical direct gap semiconductors) shows a strong, abrupt and concave absorption edge at the band gap^27,28^. On the other hand, both indirect as well as direct forbidden transitions (so-called pseudo-direct gap) show a small offset and a slow, convex-shaped increase in energy^27,29,30^. In addition, indirect gap transitions require phonon emission and therefore, as pseudo-direct transitions, should show much weaker absorption than direct allowed transitions. With that in mind, the results reported in Fig. 2a can be understood as a direct observation of the pseudo-direct band gap nature of the WZ phase in GaP.

In order to analyze in more detail the absorption edges of the PLE spectra, it is important to compare the experimental data with available theoretical results^4–6,12^. Belabbes and Bechstedt^12^ have predicted the WZ GaP band gap at 2.12 eV and an almost forbidden fundamental transition, which would behave as a pseudo-direct band gap semiconductor. Analyzing the reported theoretical results of the wave vector dependence of the optical transition matrix element, it is clear that the dipole matrix element corresponding to the fundamental transition is non-null at the Γ-point, but is very small for the wave function perpendicular to the c-axis and it increases only for a certain wave vector k, as reported by Belabbes and Bechstedt^12^. This is consistent with the quite weak absorption band observed around the gap (band *A*), at 2.19 eV. Belabbes and Bechstedt^12^ also suggested that the optical transitions observed in the previous experimental data do not strictly follow k-vector conservation, since the disorder introduces non-conservation of k. A similar analysis can be carried out for the absorption bands *B* and *C*, for which the expected matrix element at k = 0 is also negligible. However, much stronger absorption is observed for these bands as compared to the band *A*, indicating that this model cannot explain all absorptions. Furthermore, the theoretical description by Belabbes and Bechstedt^2,12^ provides valence band splitting values predicted as Δ_AB_ = 42.5 meV and Δ_BC_ = 132.5 meV. Therefore the splitting between *A* and *B* bands is smaller than between *B* and *C*, contrary to the experimentally observed behavior, i.e., Δ_AB_ > Δ_BC_ with Δ_AB_ = 110 meV and Δ_BC_ = 30 meV reported here.

In order to clarify this discrepancy, we have calculated the band structure of WZ GaP phase using DFT implemented in the VASP code with the HSE06 functional, which provides a realistic band gap ordering. Figure 2c shows that the predicted band gap energy was 2.10 eV, which is close to the previously calculated^4,12^ and measured results^2,7^, as well as to the data presented here. Also, the calculated energy splitting between the three highest valence bands were 230 meV and 110 meV, respectively. These values, despite being overestimated, are in qualitative agreement with our experimental data (Δ_AB_ > Δ_BC_).

Moreover, our model provides an inversion between the conduction bands; the lowest energy conduction band, in this case, is the Γ_7_ band with p-orbital contribution, while Γ_8_ s-orbital shows the highest energy. In this case, the measured *A*, *B* and *C* absorption edges should correspond to the transitions involving Γ_9_, Γ_7+_, Γ_7-_ valence bands and Γ_7_ conduction band. Regardless of this band inversion, the three lowest energy electronic transitions are still dipole forbidden at the center of the Brillouin zone. Therefore, the partial breakdown of the selection rules – usually related to the presence of impurities and crystal defects - is required to explain the transition observed here. In our case, PL spectra (inset in Fig. 2a) clearly shows that impurities and/or point defects are present in the material, leading to broad band emissions below 2.0 eV. Thus, the excess of these impurities may lead to the partial breakdown of the k-selection rules. It is important to notice that Belabbes and Bechstedt^12^ also explored this possibility in their models; however, the qualitative agreement for the experimental valence band splitting behavior presented here indicates that the electronic band structure shown in Fig. 2c may provide a better description of the absorption data of Fig. 2a,b. Nevertheless, the exact distinction between the two electronic band structure models is not possible from the optical absorption data alone and it is out of the scope of the present work.

**Figure 3:**
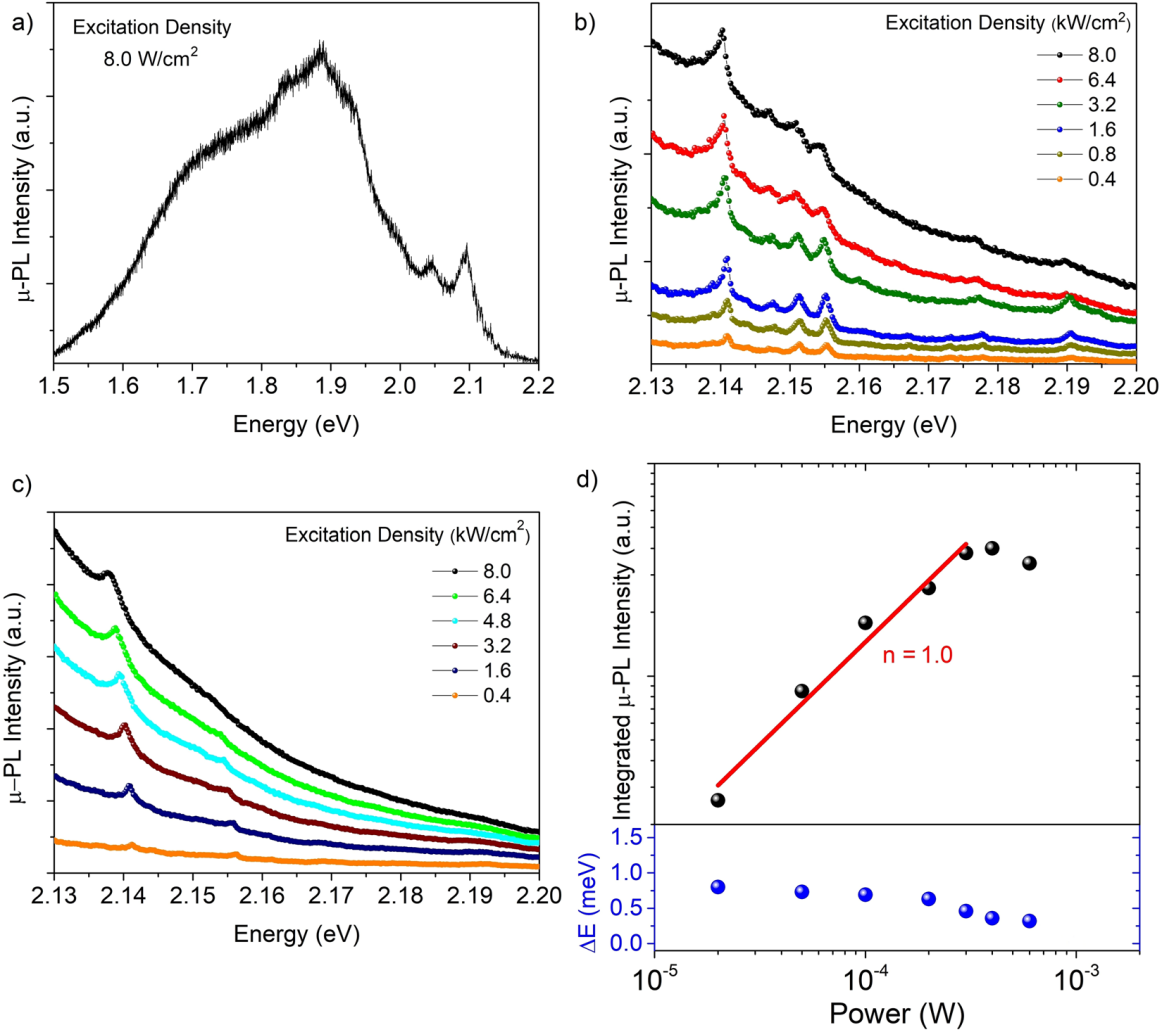
(**a**) µ-PL spectrum acquired at the tip of the GaP structure at 5 K under low excitation density (8.0 W/cm^2^) regime. (**b**) and (**c**) 5K µ-PL spectra under different excitation densities in the high power regime (≥ 0.1 kW/cm^2^). The two set of spectra were acquired at different positions on the GaP structure, at the tip and in the body segment, respectively. (**d**) Integrated µ-PL intensity and energy shift of the peak at 2.14 eV as function of the excitation power.

Low temperature (5 K) µ-PL results also reveal the optical properties of our GaP structure. The micro-PL spectrum depicted in Fig. 3a, acquired at the tip of the GaP structure under low excitation density, shows that the luminescence is dominated by emissions below 2.0 eV, associated with deep levels from residual impurities or defects, and two emission lines at 2.04 eV and 2.09 eV, associated with DAPs^1,2,10^. This behavior is similar to the macro-PL in Fig. 2a (inset); the same overall spectrum is observed for different segments along the structure, with small variation in relative intensities.

Increasing the excitation density over three orders of magnitude, additional sharp peaks in the range of 2.13 eV – 2.22 eV appear, see Fig. 3b. Interestingly, the peaks at 2.140 eV and 2.155 eV remain constant when measurements are acquired at different regions along the GaP structure, as we can see in Fig. 3b,c, which shows the μ-PL spectra versus excitation density measured on different positions, at the top and the basis, respectively. It is clear that other peaks appear or disappear at different energies when the μ-PL is carried out on different positions along the GaP structure. Assali *et al*.^3^ have attributed the sharp lines observed in that spectral range to quantum confinement effects around stacking fault defects. However, the peaks at 2.140 eV and 2.155 eV, which are present in the whole GaP structure, rapidly saturate under high excitation power, as shown in Figs. 3c and 3d. Therefore, these two emissions should not originate from locally formed crystalline defects, and are representative of all WZ GaP structure, with a distinct origin.

Careful analyzes of the emission at 2.140 eV shows that this peak has a FWHM of ~ 1 meV (limited by our system resolution) and increases in intensity with excitation density following a power law with exponent n = 1.0 (Fig. 3d), typical for excitonic recombination^31^. Moreover, the emission seems to saturate, indicating bound excitons, which are in agreement with the previous results of Assali *et al*.^2^

On the other hand, the peak at 2.155 eV saturates very quickly with excitation power; the background generated from the deep level emissions dominates and the peak is smoothened out. This peak is also attributed to a bound exciton. It is interesting to note that, in the PL spectra of Fig. 3b, additional sharp emissions appear below ~ 2.155 eV and are equally spaced within ~ 4 meV. Similarly, the emission at 2.19 eV is also accompanied by two very weak emissions separately by ~ 10 meV. This energy separation and the local dependence of all these minor peaks suggest that they are related to stacking faults; these defects create quantum wells of ZB monolayers in the WZ matrix^3^. Therefore, their emission energies depend on the number of zinc blend (ZB) monolayers in each well, so that emissions are separated by multiples of ~ 5 meV.

## Conclusions

In summary, we have investigated the optical properties of GaP nanowires by growing GaP structures with large volumes in the hexagonal phase. The absorption edges of this material were measured, providing experimental evidence for WZ GaP band structure with three valence band splitting. Direct evidence that the bandgap is pseudo-direct with a band gap of (2.19 ± 0.02) eV at low temperature was also demonstrated, along with bound-excitonic recombination at 2.14 eV. DFT calculations provide support for PLE spectra taking place at the Γ point. Thus, our results improve the knowledge concerning the basic electronic band structure parameters of this relatively new material.

## Supplementary information

Supplementary Information.

## Data Availability

The datasets generated during and/or analyzed during the current study are available from the corresponding author on reasonable request.
