# Author Correction: Optical Absorption Exhibits Pseudo-Direct Band Gap of Wurtzite Gallium Phosphide

**DOI:** 10.1038/s41598-020-67918-2

**Published:** 2020-07-09

**Authors:** Bruno C. da Silva, Odilon D. D. Couto, Hélio T. Obata, Mauricio M. de Lima, Fábio D. Bonani, Caio E. de Oliveira, Guilherme M. Sipahi, Fernando Iikawa, Mônica A. Cotta

**Affiliations:** 1 Institute of Physics “Gleb Wataghin”, University of Campinas, Campinas, SP 13083-859 Brazil; 2 Materials Science Institute, University of Valencia, 22085, 46071 Valencia, Spain; 3 São Carlos Institute of Physics, University of São Paulo, 369, São Carlos, SP 13566-590 Brazil

Correction to: *Scientific Reports* 10.1038/s41598-020-64809-4, published online 13 May 2020

The Acknowledgements section in this Article is incomplete.

“We acknowledge the Brazilian Nanotechnology Laboratory (LNNano/CNPEM) for granting access to their electron microscopy facilities, and the Brazilian agency CAPES. B. C. da Silva acknowledges FAPESP for his scholarship (grant 15/24271-9). We also thank M. M. Tanabe for technical support with the optical setup. This work was financially supported by the Brazilian agencies CNPq (grants 305769/2015-4, 432882/2018-9, 479486/2012-3 and 441799/2016-7) and FAPESP (grants 15/16611-4, 12/11382-9 and 16/16365-6).”

should read:

“We acknowledge the Brazilian Nanotechnology Laboratory (LNNano/CNPEM) for granting access to their electron microscopy facilities, and the Brazilian agency CAPES. B. C. da Silva acknowledges FAPESP for his scholarship (grant 15/24271-9). We also thank M. M. Tanabe for technical support with the optical setup. This work was financially supported by the Brazilian agencies CNPq (grants 305769/2015-4, 432882/2018-9, 479486/2012-3, 441799/2016-7, 408916/2018-4 and 308806/2018-2), CAPES-CsF (grant No. 88881.068174/2014-01) and FAPESP (grants 15/16611-4, 12/11382-9 and 16/16365-6).”

