# Supplementary material for: Optical Absorption Exhibits Pseudo-Direct Band Gap of Wurtzite Gallium Phosphide
Source: Sci Rep. 2020 May 13;10:7904. doi: 10.1038/s41598-020-64809-4 (PMC7221080; doi:10.1038/s41598-020-64809-4)
Supplement: Supplementary file 1 — Supplementary Information. [file 41598_2020_64809_MOESM1_ESM.docx]

Supplementary Information

Optical Absorption Exhibits Pseudo-Direct Band Gap of Wurtzite Gallium Phosphide

Bruno C. da Silva^1^, Odilon D. D. Couto Jr.^1^, Hélio T. Obata^1^, Mauricio M. de Lima^2^, Fábio D. Bonani^3^, Caio E. de Oliveira^3^, Guilherme M. Sipahi^3^, Fernando Iikawa^1^ and Mônica A. Cotta^1^

*1 Institute of Physics “Gleb Wataghin”, University of Campinas, 13083-859 Campinas, São Paulo, Brazil*

*2 Materials Science Institute (ICMUV), University of Valencia, 22085, E-46071, Valencia, Spain*

*3 São Carlos Institute of Physics, University of São Paulo, 369, 13566-590 São Carlos, SP, Brazil*

**SI.1 - Determination of the optical absorption energies**

Dipole-forbidden (or pseudo-direct) transitions are characterized by the following absorption response:^1^

$$\alpha=A{(E-E_{g})}^{3/2}$$

We can plot a linear relation with energy by:

$$\alpha^{2/3}=A\left( E-E_{g} \right)=aE+b$$

Therefore,

$E= E_{g}$ , when $\alpha^{2/3}= 0$

$$E_{g}= \frac{-b}{a}$$

Direct dipole allowed transitions exhibit a different response^1^, $\alpha=A\left( E-E_{g} \right)^{1/2}$. However, no linear curves could be reliably fitted to the data using the exponent $n=1/2$; the best linearization was obtained with this specific exponent ($n=3/2$). The absorption background generated due to impurity absorptions, I_0_, has been subtracted from the optical data before linearization of the first absorption edge, Fig. S1.


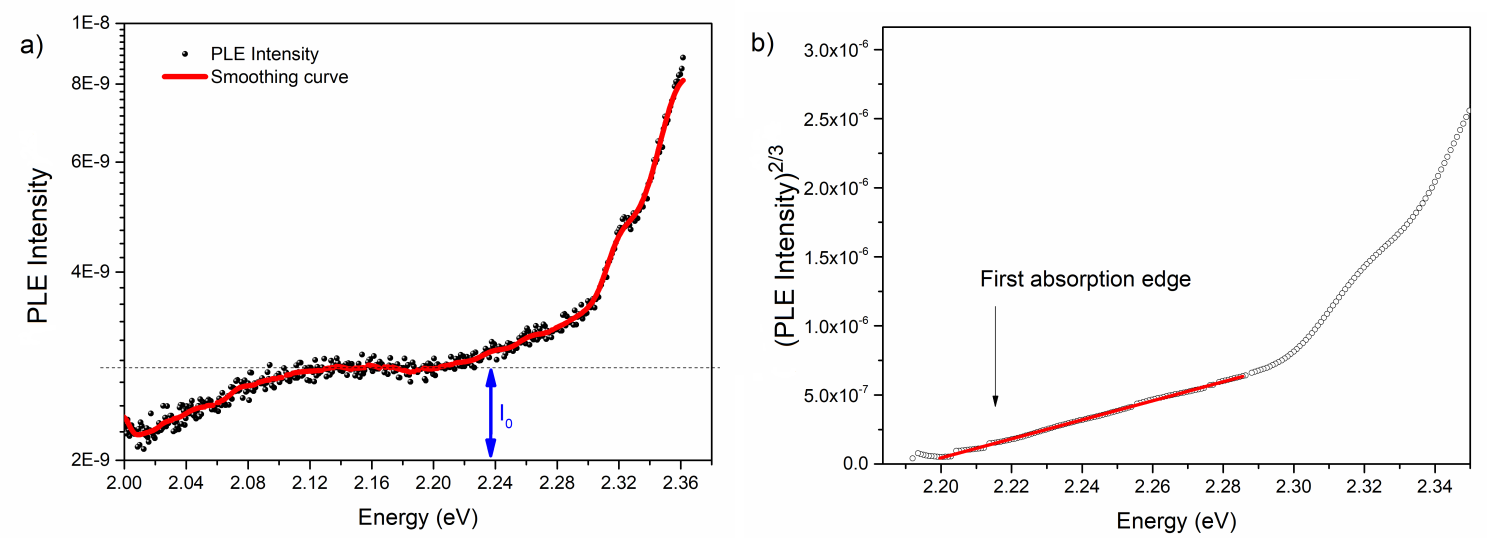


**FigureS1–** (a) PLE spectrum and smoothing curve. The I_0_ background, subtracted for the first absorption edge, is highlighted. (b) Linear PLE intensity and linear fit for the first absorption edge.

The linear fitting for the first absorption edge (Fig. S1b), associated to the band gap, provides:

| **Parameters** | **Value (10^-6^)** | **Uncertainty (10^-8^)** |
| --- | --- | --- |
| a | 6.893 eV^-1^ | 2.31 eV^-1^ |
| b | -15.12 | 5.18 |

Therefore,

$$E_{g}=\frac{-b}{a}=2.194 eV$$

The uncertainty $\mu_{linear fit}$ was:

$$\mu_{linear fit}= E_{g}\sqrt{\left( \frac{\mu_{a}}{a} \right)^{2}+\left( \frac{\mu_{b}}{b} \right)^{2}}=12.3 meV$$

The propagation of the uncertainty provides:

$$\mu_{E_{A,B,C}}^{2}=\mu_{linear fit}^{2}+\mu_{single monoch.}^{2}+\mu_{others}^{2}$$

Where $\mu_{linear fit}$ is the uncertainty of the linear fitting of the considered absorption edge (Fig. S1b), $\mu_{single monoch.}$ is the uncertainty of the single monochromator coupled to the Xe lamp, which was used as excitation source, and $\mu_{others}$ is an uncertainty related to the double monochromator used for the detection, background subtraction and other less significant sources. The energy uncertainty for the double monochromator is ~ 1 meV; considering that $\mu_{others}$ << $\mu_{single monoch.}$ and $\mu_{others}$ << $\mu_{linear fit}$:

$$\mu_{E_{A,B,C}}^{2}=\mu_{linear fit}^{2}+\mu_{single monoch.}^{2}$$

$$\mu_{E_{A,B,C}}=\sqrt{\mu_{linear fit}^{2}+\mu_{single monoch.}^{2}}$$

We have measured the uncertainty related to the single monochromator that is coupled to the Xe lamp as:

$\mu_{single monoch.}= 8 meV$

For the first absorption edge, related to the band gap $E_{A}=E_{g}$:

$$\mu_{E_{g}}= \sqrt{\left( 12.3 meV \right)^{2}+\left( 8 meV \right)^{2}}\cong0.0147 eV$$

$$\mu_{E_{g}}\cong0.015 eV=20 meV$$

Therefore,

$$E_{g}=(2.19 \pm0.02) eV$$

The other two lowest electronic transitions have also been predicted to be dipole-forbidden transitions. Thus, applying the same analysis for each absorption edge, we have found:

$$E_{B}=\frac{-b}{a}=2.298 eV$$

$$E_{C}=\frac{-b}{a}=2.329 eV$$

and

$$\mu_{E_{B}}= \sqrt{\left( 23.8 meV \right)^{2}+\left( 8 meV \right)^{2}}\cong0.0251 eV$$

$$\mu_{E_{C}}= \sqrt{\left( 18.5 meV \right)^{2}+\left( 8 meV \right)^{2}}\cong0.0202 eV$$

$$\mu_{E_{B}}\cong0.025 eV\cong30 meV$$

$$\mu_{E_{C}}\cong0.020 eV=20 meV$$

Therefore,

$$E_{B}=(2.30 \pm0.03) eV$$

$$E_{C}=(2.33 \pm0.02) eV$$

References

1. Yu, P. Y. & Cardona, M. *Fundamentals of Semicondutors*. (Springer, 2010).
